# Malignant Mesothelioma, BAP1 Immunohistochemistry, and VEGFA: Does BAP1 Have Potential for Early Diagnosis and Assessment of Prognosis?

**DOI:** 10.1155/2017/1310478

**Published:** 2017-09-11

**Authors:** Emily Pulford, Kalyani Huilgol, David Moffat, Douglas W. Henderson, Sonja Klebe

**Affiliations:** ^1^Department of Anatomical Pathology, Flinders University of South Australia, Bedford Park, SA 5042, Australia; ^2^Department of Surgical Pathology, SA Pathology, Flinders Medical Centre, Bedford Park, SA 5042, Australia

## Abstract

Malignant mesothelioma (MM) is an aggressive malignancy of the serosal membranes. Early diagnosis and accurate prognostication remain problematic. BAP1 is a tumour suppressor gene commonly mutated in MM. Germline BAP1 mutation has been associated with early onset and less aggressive disease compared with sporadic MM. Sporadic BAP1 mutations are common and are associated with improved survival in MM, contrary to other malignancies. This study investigated the prognostic role of BAP1 in matched cytology and surgical specimens and aimed to investigate the association between BAP1 and the established prognostic marker VEGFA from a cohort of 81 patients. BAP1 mutation was found in 58% of histology and 59% of cytology specimens. Loss of BAP1 expression in both surgical and cytology specimens was significantly associated with poorer survival in a multivariate analysis when controlling for known prognostic indicators. Increased levels of VEGFA in pleural effusions were associated with poor survival. We conclude that the prognostic significance of BAP1 mutations in MM cannot be determined in isolation of other prognostic factors, which may vary between patients. Pathologists should employ caution when commenting on prognostic implications of BAP1 status of MM patients in diagnostic pathology reports, but it may be useful for early diagnosis.

## 1. Introduction

Malignant mesothelioma (MM) is an aggressive malignancy of the serosal membranes and is attributable in most cases to prior asbestos exposure. Prognosis is currently poor, and most deaths occur within 12–18 months of diagnosis, with even shorter survivals on average for pleomorphic, sarcomatoid, and desmoplastic MMs. Most diagnoses occur at a late stage, related to nonspecific symptoms and the long latency period of the disease, and treatment response is limited. Established prognostic indicators such as histological subtype, age, and gender can give some insight into predicting patient survival [1–4]; however, there are few definitive and specific prognostic indicators routinely used to predict likely outcomes for individual patients. Previously identified additional prognostic indicators include levels of vascular endothelial growth factor (VEGF), mesothelin and fibulin-3 in serum and pleural effusion, or expression of aquaporin-1 in tissue [5–10]; however, these are not routinely used in clinical decision-making. Identification of robust prognostic markers may allow for individualised patient management regimens, with improved patient survival and quality of life.

The tumour suppressor gene BRCA-associated protein 1 (BAP1) is located at 3p21 and is commonly mutated in MM tissue samples [11–17]. BAP1 is localised to the nucleus and functions as a deubiquitinating enzyme, specifically regulating chromatin remodelling, functioning as a mediator of DNA damage responses and growth suppression [18–20]. Recent evidence has shown that BAP1 plays a role in modulation of calcium-induced apoptosis, and consequently mutation may result in accumulation of DNA-damaged cells and greater susceptibility to development of malignancy [21]. Tumour suppressor gene function was previously thought to be dependent on nuclear localisation; however, recent evidence also suggests cytoplasmic activity [18, 21, 22]. Most published mutations result in a truncated protein or mRNA decay, and the site of the mutation has no known association with the resulting cancer type [23, 24]. BAP1 knockdown *in vitro* has resulted in decreased cell proliferation and mediation of apoptosis in MSTO211H, HMeso, and H2373 mesothelioma cell lines, and reintroduction of wild-type BAP1 in BAP1-null cell line NCI-H226 promoted cell growth, yet another study reported that this was counterbalanced by increased apoptosis, indicating that the consequences of *in vitro* manipulation may be cell type dependent [11, 22]. Additionally, *in vivo* evidence in nude mice has shown that injection of NCI-H226 (BAP1 negative) cells confers less tumorigenicity when these cells are infected with lentivirus carrying wild-type BAP1 compared to the mutated BAP1 [22], further indicating its role in tumour suppression. Mutations of BAP1 may be sporadic or familial. Germline mutations in BAP1 have been observed in families with a high frequency of MM, associated with earlier age of onset, among other malignancies such as uveal, ocular, and cutaneous melanoma [16, 23, 25–27]. Given the high incidence of asbestos exposure in previous decades, with incidence of MM still rising in some nations, a better understanding of potential predisposing genetic factors may provide insight into why MM develops in only a minority of asbestos-exposed patients and may allow identification of exposed persons at risk of developing MM.

The molecular pathways of BAP1 in carcinogenesis are not well understood, and it was previously suggested that BAP1 interacted with BRCA-1; however, recent evidence has contradicted this theory [28]. There is evidence of interaction with HCF-1 and subsequent regulation of cell proliferation at the level of the cell cycle G_1_/S checkpoint as well as apoptosis regulation; however, the impact of upregulation or downregulation of BAP1 is dependent on cell type [11, 22]. A number of associated mutations have been identified as candidate genes in MM, particularly those involved with regulation of hypoxia-inducible factor (HIF1*α*) and VEGF [29–31]. HIF1*α* is regulated by VHL, which along with BAP1 is commonly mutated in renal cell carcinoma and located on the short arm of chromosome 3 [27]. Mutations in VHL have been detected in MM samples [32], along with other mutations at 3p21 such as SETD2 and PBRM1 [33].

Initially, acquired BAP1 mutation frequency in MM was underestimated due to the use of Sanger sequencing and NGS, which missed larger deletions [33, 34]. Consequently, immunohistochemistry is thought to be the most reliable method for detection of BAP1 mutation. Loss or mutation of BAP1 can be visualised in most cases as undetectable nuclear immunoreactivity in immunohistochemistry studies [11]. Loss of BAP1 expression has been identified as an adverse prognostic indicator in a number of different cancers [35–38], potentially related to its role in tumour suppression. In MM however, loss of BAP1 expression detected by immunohistochemistry has shown contradictory prognostic implications, with some studies reporting improved patient outcomes with loss of BAP1 expression, suggesting a protective mechanism in disease development [14–16], and others reporting no change in patient survival [39–41]. This, in conjunction with germline data, signifies potential for its involvement in disease pathogenesis.

This study recruited a cohort of MM patients diagnosed at Flinders Medical Centre and established BAP1 status by immunohistochemistry on matched surgical and cytology specimens (i.e., samples were taken at the same time). We aimed to evaluate any prognostic significance of BAP1 status within this cohort, independent of established prognostic indicators, in order to compare our findings with previous contradictory data. This may provide further insight into disease pathogenesis and allow for more specific prognostic predictions in pathological analysis.

## 2. Methods

### 2.1. Patient Recruitment

A cohort of 83 patients was recruited, diagnosed at the Department of Anatomical Pathology at Flinders Medical Centre between the years of 2006 to 2015. Archival tissue and cytology blocks were available through SA Pathology. Patients were included on the basis of histological diagnosis of MM, availability of adequate tissue and cytology blocks, and clinical follow-up information. Additionally, 18 cytology specimens from patients presenting with malignant effusions due to metastatic lung adenocarcinoma were also included to compare BAP1 expression between MM and adenocarcinoma, which may be useful for differential diagnoses in selected cases. This work was approved by the Southern Adelaide Clinical Human Research Ethics Committee (approval number 381.09).

### 2.2. Pleural Effusion Specimen Collection

Pleural effusion samples were obtained through SA Pathology after processing for diagnosis. Samples were spun at 500*g* for 5 min, and the supernatant was stored at −80°C immediately until further use. Of this cohort, 18 samples were available for processing.

### 2.3. Immunohistochemical Analysis

Paraffin sections were cut at 0.4 *μ*m thick, then deparaffinised, and rehydrated in graded concentrations of xylene and ethanol. Slides were immersed in 3% H_2_O_2_ in 50% ethanol to quench endogenous peroxidases. Heat-induced alkaline retrieval was performed using Dako Target Retrieval Solution. Sections were blocked with 10% normal goat serum in TBS for 30 mins. Sections were coated with 1 : 3000 mouse monoclonal BAP1 antibody (Santa Cruz Biotechnology, Texas, USA, sc-28383) and were incubated at 4°C overnight. The Novolink Max Polymer Detection kit (Leica Biosystems, Nussloch, Germany) and DAB^+^ Chromogen System (Dako Australia Pty. Ltd., NSW, Australia) were used for detection, before haematoxylin counterstaining. Diagnostic clinical procedures related to diagnosis of the cases were performed in a NATA-approved laboratory using QAP validated tests.

### 2.4. Histological Scoring

All slides underwent blinded assessment by two RCPA-qualified pathologists, S.K. and D.M. A positive result was defined as positive nuclear labelling in any number of tumour cells, regardless of background cytoplasmic reactivity. Cytology specimens were excluded if cell count was not satisfactory (>20 malignant cell/section required).

### 2.5. VEGFA ELISA

Levels of VEGFA, an established prognostic factor in MM, were correlated with survival to assess potential interaction of BAP1 and VEGFA. VEGFA levels in pleural effusions were tested on the 17 patients for whom effusion fluids were available. The VEGFA Duoset ELISA (DY293B, R&D Systems) was used to detect VEGFA levels as per kit instructions. Concentration was determined via comparison to standard curves.

### 2.6. Statistical Analysis

Kappa scores were obtained to analyse agreement between pathologist opinions. Survival was calculated as the number of months between diagnosis and death of the patient, or last follow-up in the case of patients still alive. If a patient was still alive at the last follow-up, survival was censored. Kaplan-Meier curves were implemented to analyse survival, and Cox Regression univariate and multivariate analysis were used to evaluate covariates for statistical significance. A *p* value of <0.05 was deemed statistically significant. Age, sex, and histological subtype were entered into a multivariate model as they are universally accepted prognostic factors in MM.

## 3. Results

### 3.1. Patient Characteristics

In total, 81 patients were eligible for analysis of surgical sections, and 57 of these patients had accompanying cytology sections for analysis.  Table 1 summarises the general patient characteristics for the entire cohort. Mean survival was 10 months, and 98% of patients were deceased at the conclusion of the study. Histological subtype, age, sex, and chemotherapy are known prognostic indicators in MM patients, with the epithelioid subtype generally having better survivals than sarcomatoid and biphasic MMs. This was demonstrated in the multivariate analyses of the surgical cohort, where the epithelioid subtype showed a median overall survival of 11.2 months, significantly greater than that of the sarcomatoid subtype (7.8 months, *p* = 0.015). Chemotherapy and conservative therapy (pleurodesis and palliative care) were the most common treatment options but were not significant prognostic factors in univariate or multivariate analysis. Age and sex had no statistical impact on survival.

### 3.2. Immunohistochemistry

Positive nuclear immunoreactivity in tumour cells of surgical and cytology specimens was considered as retained BAP1 expression. Cytoplasmic expression was disregarded. Agreement between pathologist assessment (*κ* = 0.88) and surgical versus cytology BAP1 status (*κ* = 0.85) for matched patients was analysed, both of which indicated good agreement. BAP1 expression was lost in 61% of epithelioid, 62% of biphasic, and 36% of sarcomatoid histology specimens (Table 2). Similarly, BAP1 expression was lost in 63% of epithelioid, 57% of biphasic, and 43% of sarcomatoid cytology specimens. Negative BAP1 labelling was observed in 62% of male and 44% of female patient histology specimens and 65% of male and 36% of female patient cytology specimens. However, we noted that two earlier nontime-matched effusions for BAP1-negative patients that at the time were diagnosed as reactive showed loss of BAP1 when labelled retrospectively. None of the 18 metastatic lung adenocarcinomas showed loss of nuclear labelling for BAP1.

### 3.3. BAP1 Prognostic Significance

**Figure 1:**
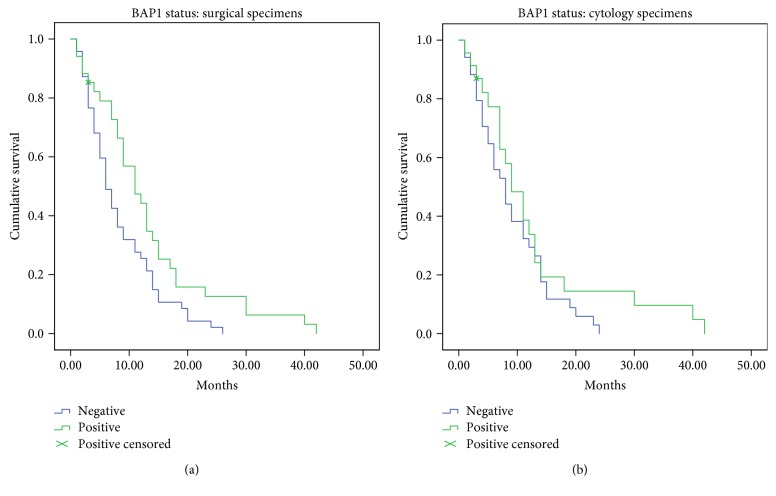
Kaplan-Meier curves for BAP1 expression in patient cohort. Retained nuclear expression in surgical sections was associated with increased survival times (a). Median survival was 6 months (*n* = 47) and 11 months (*n* = 34) for negative and positive BAP1 expression, respectively (*p* = 0.015). BAP1 expression in cytology sections was not significantly associated with prognosis (b). Median survival was 8 months (*n* = 33) and 9 months (*n* = 23) for negative and positive BAP1 expression, respectively (*p* = 0.205).

BAP1 expression status according to clinical data is summarised in Table 2. In univariate analysis, loss of BAP1 expression in surgical sections was significantly associated with poor survival within this cohort (*p* = 0.014, Table 3). In the multivariate Cox regression model, loss of BAP1 expression in surgical and cytology sections was significantly associated with poorer survival (*p* = 0.003 and *p* = 0.04, resp., Table 4). For the surgical sections, median survival times were 6 months (95% CI, 4.3–7.7 months) and 11 months (6.9–15.1 months) for negative and positive BAP1 expression, respectively (Figure 1(a)). Median survival times for cytology sections alone were 8 months (95% CI, 5.6–10.4 months) and 9 months (5.7–12.3 months) for negative and positive BAP1 expression, respectively (Figure 1(b)). In epithelioid cases alone, BAP1 loss in histology sections was associated with adverse prognostic outcomes (*p* = 0.038); however, this was not evident in analysis of other subtypes or in the cytology group.

### 3.4. VEGFA Survival Validation

**Figure 2:**
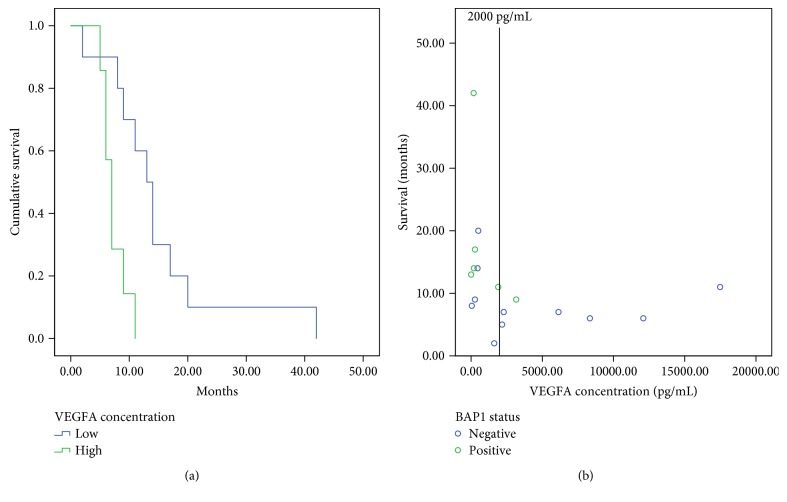
High VEGFA levels are associated with poor survival in a subsection of this cohort. All patients were diagnosed with epithelioid MM. VEGFA levels were categorised into low (<2000 pg/mL) and high (≥2000 pg/mL). Adverse prognostic outcomes for patients with high VEGFA concentrations were confirmed in this cohort (*p* = 0.005) with median survival of 13 (95% CI, 10–16.1 months) and 7 (95% CI, 5.8–8.1 months) months for high and low VEGFA concentrations, respectively (a). Of the 7 patients with high VEGFA levels, 6 showed negative BAP1 expression (b).

To further investigate prognostic implications within our cohort and investigate the potential role of BAP1 in regulating VEGFA levels, we explored pleural effusion VEGFA protein concentrations (Figure 2). Of this cohort, 6 BAP1-positive patients and 11 BAP1-negative patients had pleural effusion supernatant available for analysis. All those patients were diagnosed with epithelioid MM. VEGFA levels were categorised into low (<2000 pg/mL) and high (≥2000 pg/mL) by previously established cut-off values [6]. As expected, ELISA data showed adverse prognostic outcomes for patients with high VEGFA concentrations (*p* = 0.005). Median survival times were 13 (95% CI, 10–16.1 months) and 7 (95% CI, 5.8–8.1 months) months for high and low VEGFA concentrations, respectively. Of the 7 patients with high VEGFA levels, 6 showed negative BAP1 expression.

## 4. Discussion

Regardless of conflicting evidence and theories on prognostication, it is evident that BAP1 is commonly mutated in MM [11, 13, 14, 22]. Our data showed that BAP1-negative immunoreactivity was associated with poor survival in this cohort. During immunohistochemical scoring, it was at times difficult to ascertain nuclear reactivity status, because the cell populations often showed heterogeneity and background cytoplasmic expression. This study used nuclear reactivity as a marker for BAP1 expression. There is evidence for BAP1 activity in the cytoplasm, and normal mesothelial cells may show nuclear and weaker cytoplasmic labelling [21, 34]; but cytoplasmic labelling in isolation indicates mutated inactive BAP1. Strong cytoplasmic labelling can be difficult to interpret as it may spill over into the nucleus. For the purposes of this study, we concentrated on the loss of nuclear labelling, consistent with previous studies [13, 14, 17]. Malignant cells can be difficult to distinguish from reactive mesothelial populations—especially problematic in effusion fluids. These aspects resulted in some discrepancy between the two pathologists. Conflicting slides were reviewed by both pathologists separately, and all were independently rescored as positive. While one other study reported a kappa value of 1 [42], our study reports a value of 0.88 on initial scoring. Although this value indicates good agreement, discrepancies may result in variation of results between studies.

**Figure 3:**
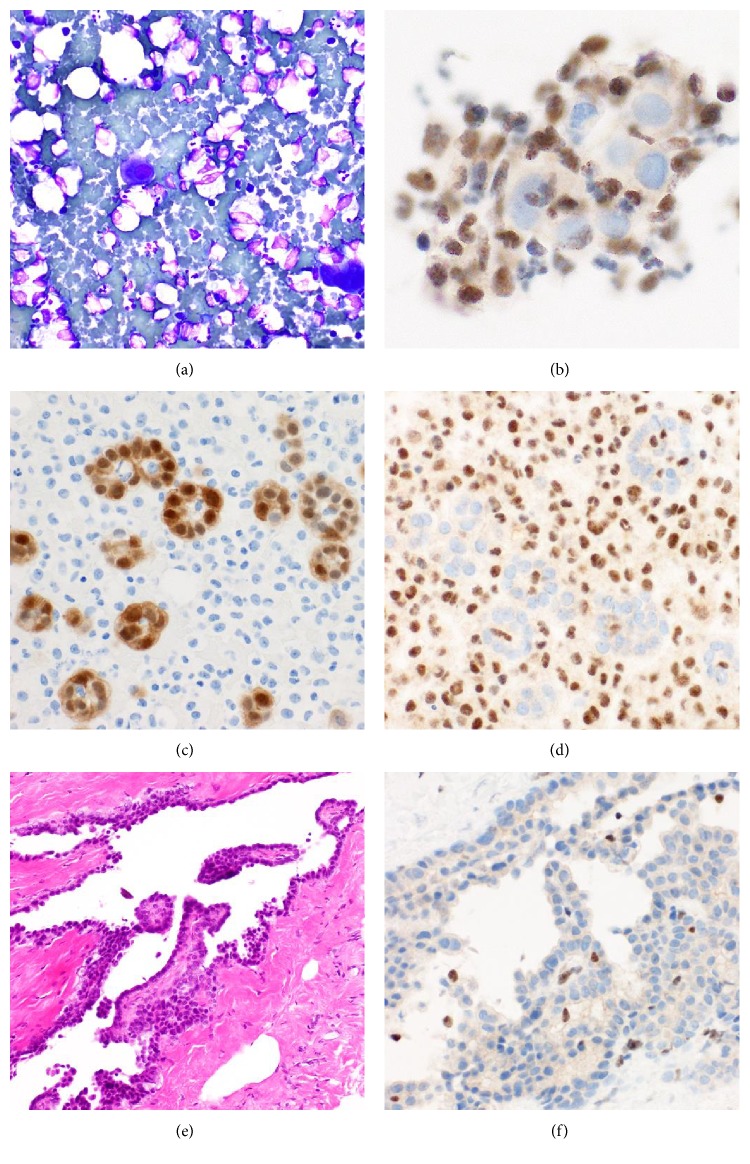
BAP1 immunohistochemistry. A patient presented with a pleural effusion that contained only scant mesothelial cells in an inflammatory background (a), diagnosed as reactive at the time. Only a few cells were seen in the cell block, but when this was labelled retrospectively for BAP 1, there was no labelling (b). Eight months later, cellular pleural effusion was drained from the same patient which contained papillary clusters of atypical mesothelial cells positive for calretinin (c) and showing loss of labelling for BAP1 (d). A diagnosis of atypical mesothelial proliferation was rendered. A concurrent pleural biopsy showed noninvasive papillary atypical mesothelial proliferation (e), also BAP1 negative (f) and in keeping with mesothelioma in situ.

The inclusion criteria of this study required at least 20 cells to be present in a matched cytology specimen for this analysis. Most cytology preparations suitable for analysis are from patients with the epithelioid subtype, as sarcomatoid subtypes generally exhibit less shedding of cells into the pleural space [43, 44]. In this cohort, 59% of MM patients showed loss of BAP1 expression in cytology analysis, and a kappa value of 0.85 between histology specimens and the corresponding cytology specimens was reported. The three cytology specimens that did not correlate with expression in histology were all of epithelioid subtype and were scored as positive contrary to the negative histology score. Due to the difficulty in discriminating between reactive mesothelial proliferations and the often scarce malignant cells in cytology preparations, this is not a surprising result as interpretation is dependent on the cell population present in that particular section. It is possible that surface reactive cells were shed or cells that may have corresponded to what is recognised as in situ MM. Cytology analysis in isolation can yield confident diagnosis of MM in conjunction with radiology and clinical information, but in the absence of histology confirmation or clinical radiological data, samples are often classified as atypical or reactive mesothelial proliferations. Sensitivity of cytology alone in diagnosis of MM is variable and dependent on subtype, with one study reporting 53% sensitivity in the epithelioid subtype compared to 20% sensitivity in sarcomatoid subtypes [45], and another reporting a sensitivity as low as 27% but a specificity of 100% regardless of subtype [46]. Nonetheless, in practice, the diagnosis is not made in isolation but always in conjunction with clinical and radiological data, and/or a concurrent or subsequent biopsy. Due to the implications of BAP1 in malignancy, where loss seems to be exclusively seen in malignant lesions and despite the lack of clarity in regard to its role in MM pathogenesis, BAP1 immunohistochemistry on cytology preparations could potentially be used as a specific screening marker for malignant versus reactive pleural effusions, although retained nuclear labelling does not exclude a MM, limiting sensitivity for diagnosis of malignancy. Some of the patients recruited in this study were admitted for pleural effusion drainage long before MM was detected, and thus, this noninvasive procedure may be useful for early flagging of these patients for follow-up. One such patient is depicted in Figure 3(). When the first pleural effusion was drained, it contained only scant mesothelial cells in an inflammatory background, not considered suspicious at the time. Eight months later, a more cellular pleural effusion was drained which contained morular clusters and some papillary clusters of atypical mesothelial cells, and the features were reported as an atypical mesothelial proliferation. BAP1 was negative on that cytology sample, and a concurrent pleural biopsy showed noninvasive papillary atypical mesothelial proliferation, also BAP1 negative and in keeping with mesothelioma in situ. Immunohistochemistry performed on the first pleural effusion in retrospect showed loss of BAP1 at that time; had BAP1 labelling been performed In addition, since none of the metastatic lung adenocarcinomas showed loss of nuclear labelling for BAP1, loss of nuclear labelling may be an additional indicator supporting a diagnosis of mesothelioma.

The current study used immunohistochemistry for detection of mutation, in the absence of sequencing and/or qPCR, but this may not detect all BAP1 mutations, as reported by us in a case of MM and uveal melanoma [47] and another study in the case of uveal melanoma alone [48], where loss of heterogeneity did not result in loss of BAP1 protein expression. In addition, it has been proposed that miRNA silencing of wild-type BAP1 may occur in heterogeneous mutation, which may not be detected by immunohistochemistry. One study has reported BAP1 loss by immunohistochemistry in the presence of normal BAP1 mRNA levels, suggesting posttranslational modification or potential miRNA silencing of BAP1 [11]. However, overall immunohistochemistry has emerged as a robust tool in this context [34].

VEGFA prognostic data was utilised in this study for two reasons: firstly, it is a previously established adverse prognostic marker when elevated in pleural effusion, and secondly, it is used to investigate the possible functional relationship between BAP1 and VEGFA. Pleural effusions were available for 18 of the recruited patients and were tested in order to validate the role of VEGFA in this cohort, to confirm that our cohort conforms to validated prognostic indicators, and to explore any association between VEGFA, BAP1 status, and survival. As expected, increased VEGFA levels were associated with adverse prognosis in this cohort, and 7 of the 8 patients with high VEGFA levels were BAP1 negative. The prognostication of MM may be more meaningful when a number of markers are used in conjunction. While there has been limited evidence of a link between molecular pathways, BAP1 mutation has coincided with VHL mutation in clear cell renal cell carcinoma (ccRCC) [49]. VHL mutation has been documented in MM previously; however, it is estimated that VHL mutation is often underestimated due to epigenetic regulation [32, 50]. It has been proposed that BAP1 plays a role in sensitisation of drugs that target epigenetic regulators such as histone deacetylases (HDACs) and inhibitors of enhancer of zeste homolog 2 (EZH2) [51, 52]. Recent evidence reports that transient BAP1 mutation increases the sensitivity of MM cell lines to HDAC inhibitors, as opposed to long-term BAP1 loss where adaptation had reduced sensitivity [51]. An inhibitor of HDAC has been used in MM with little benefit, perhaps due to disregarding BAP1 status [53]. In addition, HDAC inhibitors are implicated in VEGFA regulation, and there may be possible functional indirect role for BAP1 in this context. [54, 55]. A more refined understanding of molecular pathways involving the regulatory actions of BAP1 is required to elucidate potential interactions between molecules. The use of BAP1 in conjunction with other prognostic indicators may be more clinically useful than its use in isolation.

Our study differs from others by the higher proportion of biphasic and sarcomatoid subtypes in our cohort. In addition, our cohort has a higher mean age at diagnosis in comparison to many studies, which may indicate acquired BAP1 mutation in comparison to germline mutations. Germline mutations are associated with earlier onset of disease, as opposed to the mean age at diagnosis of approximately 70 years of age, but were also associated with improved prognosis [16, 25, 56]. Therefore, the patients in the present cohort may exhibit shorter survival times than those diagnosed at an earlier age. The mechanism of MM development in patients with germline BAP1 mutation is unknown, but it has been suggested that BAP1 mutation predisposes to augmented sensitivity to asbestos-mediated carcinogenicity [57]. This cohort included some referred cases and therefore may include some unusual cases more difficult to diagnose than most encountered in everyday practice, which again may be associated with shorter survival times. A higher proportion of BAP1 loss was seen in epithelioid cases, consistent with previous studies [14, 17]. Patients diagnosed with epithelioid MM generally have a better prognosis than those diagnosed with biphasic and sarcomatoid cases [7], consistent with the present cohort where epithelioid subtype was significantly associated with improved outcomes compared to the sarcomatoid subtype in multivariate analysis. If a higher proportion of BAP1 loss occurs in the epithelioid cases, it may skew the results when not controlled for subtype. Biphasic MMs consist of both epithelioid and sarcomatoid cell proliferations, and of the 13 biphasic cases included in this study, one sample demonstrated heterogeneity in BAP1 labelling between the two cell types with the less predominant sarcomatoid cell type retaining BAP1 expression within the sample. A previous study reported loss of expression in both cell types, but with small areas of BAP1-positive spindle-like cells, and proposed that BAP1 labelling could be used to differentiate between biphasic cell populations and epithelioid populations with reactive spindled epithelioid cells for more accurate subtyping [17].

It is clear that although a number of genes have been suggested as driver mutations in MM, a single-target approach for therapy may not be the answer. Although BAP1 has been described as a common mutation in MM cases, it is unlikely that this will offer any therapeutic target due to the high mutational load with heterogeneity of genetic changes in MM cases, with resulting treatment resistance. We suggest that care must be taken when interpreting BAP1 status in diagnostic pathology reports and relating BAP1 status to prognosis in isolation. A combination of established prognostic markers could be used to assist in more accurate individual prognostication. At this stage, histology analysis is optimal for the clinical diagnosis of MM, but we hypothesise that the use of BAP1 on cytology samples may become useful in detecting malignancy at an earlier stage. The targeted investigation of patients with multiple previous effusions or clinical suspicion before surgical biopsy may permit earlier diagnosis of MM and may provide some further uses for BAP1 as a marker of malignancy. Further investigation of the functional relationship with VEGFA status may provide further insight into pathogenesis.

## Figures and Tables

**Table 1:** Patient information.

	Count (%)
Age, y, median (range)	74 (35–94)
Sex	
Male	63 (78)
Female	18 (22)
Type	
Pleural	80 (99)
Peritoneal	1 (1)
Subtype	
Epithelioid	57 (70)
Biphasic	13 (16)
Sarcomatoid	11 (14)
Treatment	
Surgery^∗^	2 (2)
Chemotherapy	23 (28)
Radiotherapy	9 (11)
BAP1 score (surgical)	
<50%	47 (58)
≥50%	34 (42)
BAP1 score (cytology)	
<50%	34 (60)
≥50%	23 (40)

^∗^Extrapleural pneumonectomy: EPP.

**Table 2:** BAP1 status in histology and cytology specimens.

BAP1 status	Surgical		Cytology	
	Negative	Positive	Negative	Positive
Total	47 (58%)	34 (42%)	34 (59%)	23 (41%)
Gender				
Male	39 (62%)	24 (38%)	30 (65%)	16 (35%)
Female	8 (44%)	10 (56%)	4 (36%)	7 (64%)
Age				
Median (range)	74 (53–92)	74 (35–91)	74 (53–92)	74 (35–91)
Subtype				
Epithelioid	35 (61%)	22 (39%)	27 (63%)	16 (37%)
Biphasic	8 (62%)	5 (38%)	4 (57%)	3 (43%)
Sarcomatoid	4 (36%)	7 (64%)	3 (43%)	4 (57%)
Location				
Pleural	47 (59%)	33 (41%)	34 (61%)	22 (39%)
Peritoneal	0	1 (100%)	0	1 (100%)

**Table 3:** Univariate analysis for prognostic indicators in MM.

Covariate	*p*
Age (relative to median age of 74)	0.877
Sex	0.325
Subtype	0.134
BAP1 status (surgical)	0.014
BAP1 status (cytology)	0.205

**Table 4:** Multivariate analysis for prognostic indicators in MM.

	Surgical		Cytology	
	HR	*p*	HR	*p*
Age				
≤74	1.0 (ref)		1.0 (ref)	
>74	1.369	0.214	1.778	0.070
Sex				
Female	1.0 (ref)		1.0 (ref)	
Male	1.3	0.376	1.701	0.378
Subtype				
Epithelioid	1.0 (ref)		1.0 (ref)	
Biphasic	2.565	0.081	2.585	0.052
Sarcomatoid	1.745	0.013	3.156	0.015
BAP1 status				
<50%	2.226	0.003	1.882	0.047
>50%	1.0 (ref)		1.0 (ref)	
